# Evaluation of Virtual Water and Water Sustainability of Dairy Production in Trentino Alto Adige (North-Eastern Italy)

**DOI:** 10.3390/ani11041047

**Published:** 2021-04-08

**Authors:** Pier Paolo Miglietta, Federica De Leo, Benedetta Coluccia, Yari Vecchio, Fabian Capitanio

**Affiliations:** 1 Dipartimento di Scienze e Tecnologie Biologiche ed Ambientali, Università Del Salento, Via per Monteroni, 73100 Lecce, Italy; 2 Dipartimento di Scienze Dell’Economia, Università Del Salento, Via per Monteroni, 73100 Lecce, Italy; federica.deleo@unisalento.it (F.D.L.); benedetta.coluccia@unisalento.it (B.C.); 3 Dipartimento di Scienze Mediche Veterinarie, Alma Mater Studiorum Università di Bologna, Via Tolara di Sopra 50, 40064 Ozzano dell’Emilia, Italy; yari.vecchio@unibo.it; 4 Dipartimento di Medicina Veterinaria e Produzioni Animali, Università di Napoli Federico II, Via Delpino 1, 80137 Napoli, Italy; fabian.capitanio@unina.it

**Keywords:** mountain environment, water footprint, dairy farming, small-scale farms, milk, butter, cheese, product designation of origin

## Abstract

**Simple Summary:**

Dairy consumption is growing, and both the Italian production and the importation of dairy products are increasing to meet demand. As a first step toward understanding the environmental impacts of water use in the expanding dairy industry, the water footprint approach was used to compute the virtual water and water sustainability of dairy system in Trentino Alto Adige, a region characterized by small-scale farms and typical production. The results highlight that dairy products can be produced with minimal potential to contribute to freshwater scarcity. However, dairy production systems vary, both in production patterns and local environmental context. The development of dairy farming systems with high consumptive water requirements should be avoided in water-stressed regions and supported in particularly suitable regions, as Trentino Alto Adige.

**Abstract:**

Dairy products play a significant role in the human diet, but they are often associated with high freshwater resource depletion. In Italy, the dairy sector represents more than 12% of the total turnover of the agri-food sector. Trentino Alto Adige is the first Italian region in terms of number of dairy farms, but it does not register a quantitatively consistent dairy production. Notwithstanding, it is characterized mostly by small-scale farms whose strengths are the Protected Designations of Origin and typical mountain productions. The present study aims at: (i) accounting for the virtual water VW of the main dairy products (milk, butter and cheese) produced in Trentino Alto Adige; (ii) estimating the renewable water resources based on the water flow assessment of the study area; (iii) assessing water sustainability comparing the virtual water consumption of the dairy sector at a regional level to the water availability. The findings show that the consumptive virtual water related to dairy production represents about 1% of the water availability in Trentino Alto Adige. Italy’s domestic dairy production is expanding to meet the growing demand, but the expansion of dairy farming in water-stressed regions should be avoided, preferring instead suitable mountain regions where small-scale farms represent a lively entrepreneurial substrate.

## 1. Introduction

Food production and consumption have been recognized as major sources of environmental impacts [1]. The challenge of meeting the dietary requirements of an increasing world population is stimulating a strong debate about the sustainability of current food production systems, especially referring to meat and dairy productions [2,3,4].

Dairy products play a significant role in human diet due to their uniqueness, desirability, and economical and nutritional value. However, these products are often associated with high environmental impacts, mostly in terms of freshwater resource depletion [5,6]. Moreover, the dairy industry is responsible for the production of wastewaters and effluents that could have a significant environmental impact due to their pollutant characteristics [7,8].

In Italy, the dairy sector represents more than 12% of the total turnover of the agri-food sector [9]. In the European milk production ranking, Italy is fifth after Germany, France, the United Kingdom, and The Netherlands, but it is the largest producer of typical Protected Designation of Origin (PDO) dairy products [10]. In fact, the Italian dairy supply chain has a strong economic importance on the national agri-food system, thanks to its high level of know-how in management, technology, and genetics. In particular, the Italian alpine area is characterized by small-scale dairy farms whose cows are fed rations based on farm-produced forage [11]. In this area, dairy farming has a strong traditional character, with farms mainly associated to cooperative dairies that produce typical and PDO [12].

In this context, considering the importance to pursue economic growth in the dairy sector, while preventing environmental damage, virtual water (VW) is emerging as a relevant sustainability indicator. It is defined as the direct and indirect amount of water that is used in the production processes of commodities during their entire life cycle [13] and can be used as a tool for sustainable freshwater management and governance [14].

In particular, virtual water sustainability assessments of livestock production systems and products have received some attention in recent years in countries such as Ireland [15], Australia [16], China [17], Germany [18], Argentina [19], New Zealand [20], and India [21]). VW assessments, through life-cycle approaches, have been conducted analyzing several livestock products and livestock-based production systems at various spatial and temporal scales, quantifying the demand for freshwater resources of the livestock sector [16,20,22,23,24]. Recently, some authors have evaluated and compared virtual water in different types of livestock farming [5,25,26,27,28]. Other studies found that water consumption is strongly influenced by the agroecological characteristics (soil, landform, climate and year type) of the farming system [29,30,31,32,33].

In order to assess the actual impact of the dairy sector on the water resources, it is necessary to take into account hydrogeological characteristics and water availability [34]. Palmieri et al. [1] assessed the environmental sustainability of the Italian mozzarella cheese production in a traditional dairy chain, using Life Cycle Assessment, a widely recognized methodology, which allows for the identification of environmental pressures, and also of dairy production [35].

Recent studies have evaluated the economic and environmental sustainability of different mountain dairy farms in Northern Italy [36,37,38]. To date, no study has assessed the sustainability of the Italian dairy sector taking into account the water availability of the area in which production is carried out.

The present work has the following main objectives: (i) to account for the VW of the main dairy products (milk, butter and cheese) produced in Trentino Alto Adige; (ii) to estimate the renewable water resources based on the water flow assessment of the study area; and (iii) to assess water sustainability comparing the virtual water consumption of the dairy sector at a regional level to the water availability.

The Common Agricultural Policy (CAP) recognizes the importance of livestock farming in mountain areas and addresses its programs towards the support of multi-functionality, with contributions, financial incentives, and, in particular, through payments deriving from agri-environmental measures of Rural Development Plans, which are more pronounced than in the past [39].

## 2. Materials and Methods

### 2.1. The Study Area

**Figure 1:**
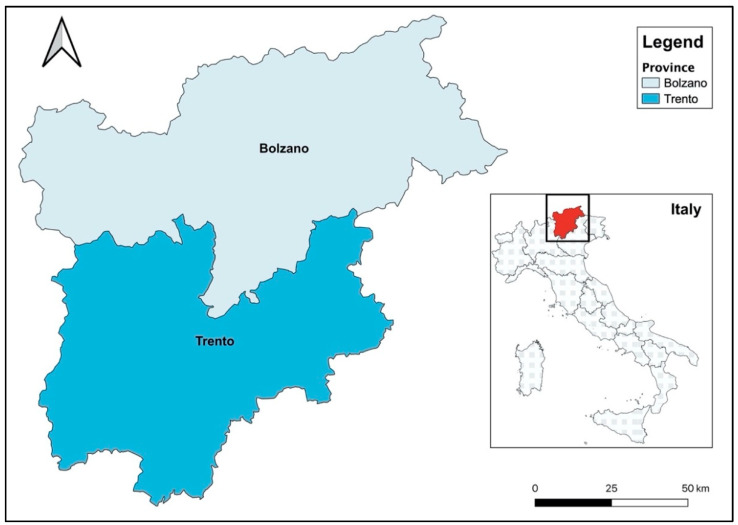
The study area.

The study was conducted in Trentino Alto Adige, an Alpine region located in North-Eastern Italy (Figure 1). Its surface extension is nearly 13,607 km^2^ and is almost entirely mountainous [40]. Forests dominate the landscape, accounting for 65% of the surface, while cultivated fields only cover 4.3% and pastures 19%. The Utilized Agricultural Area (UAA) thus amounts to 23.6% of the total. While limited in extension, farming in Trentino Alto Adige has a long historical persistence [41]. The climate is alpine, with cold and snowy winters, and short, warm summers. Precipitation is abundant: over the last 10 years, the average rainfall of the region was about 895 mm, exceeding the national average (765 mm) by about 15% [42]. The study area is crossed by numerous freshwater streams and represents the region with the highest distribution of drinking water in Italy [43].

Trentino Alto Adige is also the first Italian region in terms of number of dairy cow farms. The latter are characterized by being very small size, with an average of about nine animals per farm [10,36]. Dairy farms in Trentino Alto Adige are small-scale farms, characterized by productions typical of the mountain regions [44]. The small size of the farms has allowed for the conservation of traditional production methods, which are often economically disadvantageous because they involve a lower level of productivity and efficiency, limiting competitiveness on the national and international market [38,44]. However, contrary to the production techniques of intensive farming, dairy mountain farms are of great importance as the pasturing of animals and forage production prevents reforestation [45]. This has positive impacts on the environment and biodiversity but also ensures preservation of traditional landscapes and increases the regional tourist attractiveness [11,46]. In the study area, most of the produced milk is transformed into dairy traditional products, often recognized as Protected Designation of Origin (PDO) [47]. Although Trentino Alto Adige is not very significant quantitatively, producing only 4.5% of Italian milk, its production is mostly characterized by certified quality [48].

In particular, the mountain farming system is based on local forage resources, with a combination of fresh pasture in the summer period, and local conserved forages in the rest of the year [49]. The local forage-based diets are part of the basic link between dairy products and their original “terroir”, a notion at the basis of the PDO labelling and image of the product quality from sensory, nutritional, or safety points of view [50,51]. The forages are known to confer specific organoleptic and nutritional qualities to the milk products [52].

### 2.2. Data Sources and Description

In this section, an overview of the data inputs and sources is provided and summarized in Table 1.

Data regarding the Italian water footprint (WF) and related components for the dairy products considered in this study were extracted from Mekonnen and Hoekstra [22] (2010) and are expressed in m^3^/ton. These data, frequently used in the scientific literature [53,54,55,56,57], are based on a 10-year period of monitoring and consequently are less affected by annual weather conditions and/or climatic changes, representing the most affordable choice for macroeconomic studies.

Data about the main dairy production in Trentino Alto Adige, provided by the Italian National Institute of Statistics (ISTAT) for the year 2018, were converted into tons and assigned to the product categories established by the Harmonized System and used by Mekonnen and Hoekstra [22]. In particular, these data concern three main product macro-categories: milk, cheese, and butter. Milk was split into three further product categories based on fat content: (i) skimmed milk (containing less than 1% fat), (ii) low-fat milk (containing 1.5–1.8% fat), and (iii) whole milk (containing at least 3.5% fat). Cheese instead was split into two categories based on consistency: (i) fresh and soft cheese, and (ii) semi-hard and hard cheese.

Finally, the meteorological data were provided by the Agro-climatic Observatory of the Italian Ministry of Agriculture (MiPAAF). The weather and climate statistics used are estimated based on daily time series of meteorological stations of the National Agrometeorological network (RAN) using a non-stationary geostatistical model that takes into account the location of the stations of both the trend and the geographical correlation of climatic factors. For the purpose of the analysis, the annual precipitation and real evapotranspiration data in Trentino Alto Adige region were used.

### 2.3. Metodological and Empirical Framework

The methodology used in this analysis is divided into three different phases. The first phase consisted in identifying the water footprint values and the related component (green, blue and grey) values associated with the production of the dairy products p (skimmed milk, low-fat milk, whole milk, butter, fresh and soft cheese, and semi hard and hard cheese) in the Trentino Alto Adige region, as in the following Equation (1):

(1) $$WF\left[p\right]\;=\;WF_{Green}\left[p\right]\;+\;WF_{Blue}\left[p\right]\;+\;WF_{Grey}\left[p\right]$$

Conceptually, the surface and groundwater utilized for producing any of the selected dairy products is indicated as the $WF_{Blue}$ of the product p. The rainwater utilized for producing any of the selected dairy products p, excluding the rainwater that runs off [59], is indicated as the $WF_{Green}$ of that dairy product. The quantified amount of water theoretically required to dilute pollutants obtained along the production processes and to bring back the water quality to its acceptable standard is indicated as the $WF_{Grey}$ [59]. The sum of green and blue WFs is also called consumptive WF, while the $WF_{Grey}$ is also called degradative WF [60].

Through the evaluation of the individual WF components (green, blue, grey), this first phase aimed at highlighting quantitative differences in terms of water consumption but also at establishing the different impacts on the environment caused by the production of dairy products.

In the second phase of the methodological framework, the virtual water (VW) related to dairy products of Trentino Alto Adige region was calculated by multiplying the production volumes of each product p considered in the analysis by its associated water footprints [22]. In particular, the Italian weighted average water footprint was used, whose value takes into account grazing, industrial, and mixed farming systems. In order to calculate the virtual water volumes, the following Equation (2) was adopted:

(2) VW[p, t, c] = WF[p, c] × DP[p, t]

VW[p, t, c] denotes the virtual water (m^3^/y) related to each WF component c used to produce the quantities of product p in the year t, and DP represents the dairy production of Trentino Alto Adige in the year t.

In the last methodological phase, a measure of water availability (WA) [61] related to the study area was calculated. The available volume of water in the study area was established, analyzing the water balance and in particular the effective infiltration from precipitation. The latter can be considered as a proxy of the volume of renewable water resources, i.e., available water [61]. In fact, due to the Italian morphology and geographical features, the hydrological system can be considered as a closed water system and the precipitation as the major contributor to water availability [62].

The volume of precipitation flowing into the study area must be known in order to calculate the overall inflows and outflows. The average annual values for precipitation (P) and those of the real evapotranspiration ($ET_{r}$) were extracted from MiPAAF, allowing for the analysis of water exchange between the ground and the atmosphere.

The effective precipitation ($P_{e}$) was estimated as the difference between precipitation (*P*) and the real evapotranspiration (*ET_r_*). After having obtained values for effective precipitation (*P_e_*) and runoff (*R*), the latter estimated using the method developed by Kennessey [63], effective infiltration (*I_e_*) values were calculated, thus providing an estimation of the recharge and hence the potentially available volume of water (Equation (3)) [64].

(3) $$WA\;=\;\left(P\;-\;ET_{r}\right)\;-\;R$$

**Figure 2:**
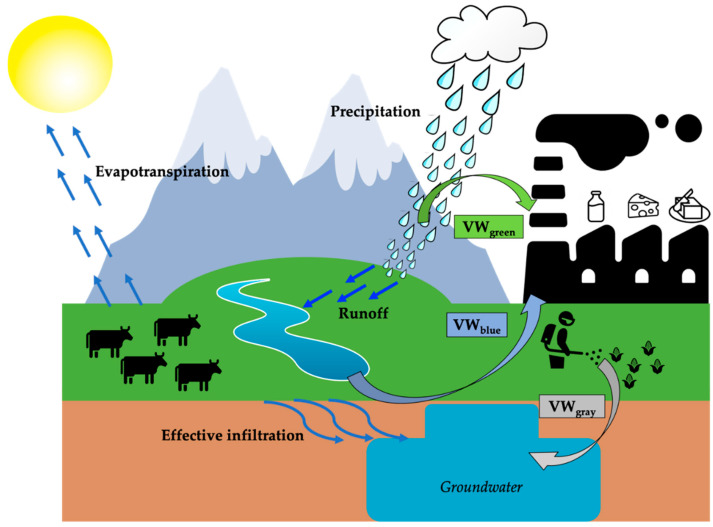
Hydrological cycle and consumptive virtual water of dairy products.

The result of Equation (3), expressed in mm, was converted to m^3^, considering the territorial extension of the study area [40]. Comparing the VW with the WA, as illustrated in Figure 2, the effective impact of dairy products on the local water resource in Trentino Alto Adige can be evaluated and the water sustainability of the dairy sector discussed.

## 3. Results

**Figure 3:**
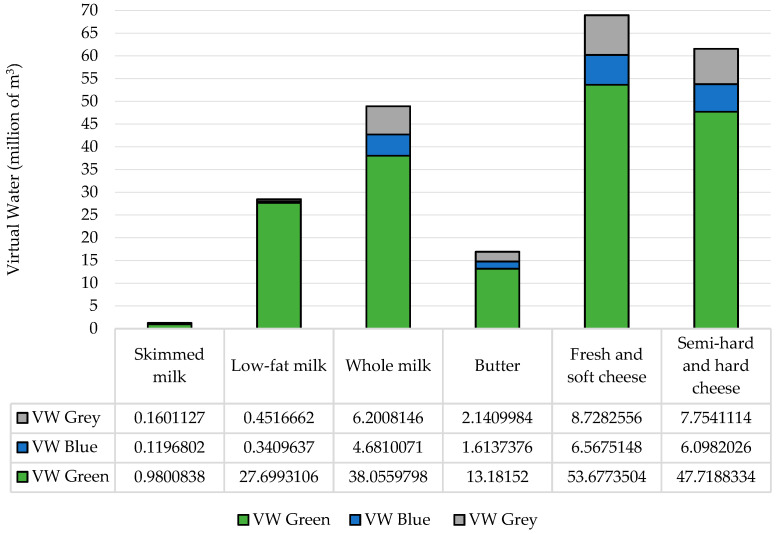
The blue, green, and grey virtual water of dairy products in Trentino Alto Adige in 2018.

The results showed that in 2018, the total virtual water of the dairy sector in Trentino Alto Adige was 326,836,622.9 m^3^, with relevant differences between the three VW components. The VW estimates for each dairy product are provided in Figure 3.

For all product categories, the green VW had the largest contribution to the total VW, while blue VW was extremely low in the production of skimmed milk, 1% of the total water, and for all the other categories did not exceed 10%. The grey VW was higher than the blue VW for all the analyzed products with greater values for butter, fresh and soft cheese, and semi-hard and hard cheese. This finding was similar to the results of Palhares and Pezzopane [27], Roibás et al. [65] and Owusu-Sekyere et al. [53], which also indicated green VW as the major component of the total VW for dairy products.

**Figure 4:**
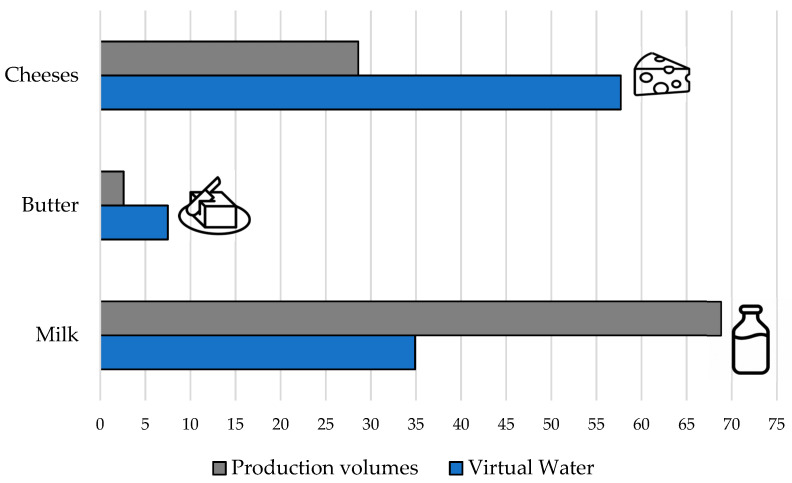
Percentage of the main product categories on the total quantity of production and on the total consumption of water in the dairy sector.

The results revealed that the highest total VWs were associated with cheese. In fact, looking at the sum of the two types of cheese considered (fresh cheese and hard and semi-hard cheese), cheese was responsible for about 60% of the virtual water consumption of the entire sector, while representing less than 30% of total production. On the other hand, although about 68% of dairy production was represented by milk (whole, partially skimmed and skimmed), its incidence in terms of virtual water consumption was extremely lower than that of cheese (Figure 4). This result was in line with previous studies that evaluated the environmental impact of cheese production compared to other dairy products, finding that the cheese supply chain had the highest demands for raw materials, energy, and water [66,67]. Cheese production processing involves numerous steps that are both time-consuming and environmentally expensive [68], which make it the main product in the dairy industry due to its greater economic value.

On the contrary, drinking milk, requiring fewer steps along the supply chain, necessitates a lower virtual water consumption (3–4% for each component) than all other dairy products.

**Figure 5:**
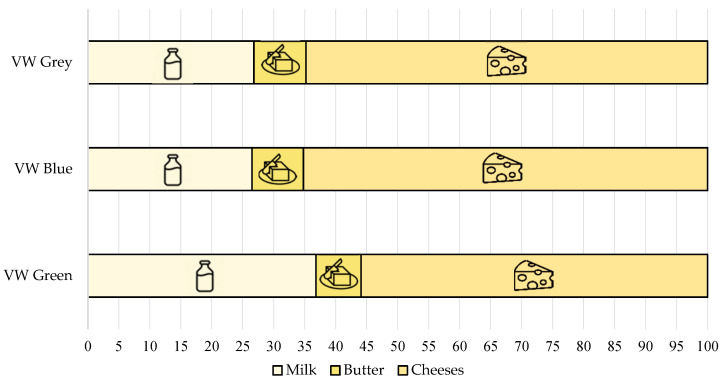
Percentage composition of virtual water of the main categories of dairy products.

Figure 5 shows that cheese had the highest green, blue, and grey VW values, highlighting a high environmental impact in terms of virtual water consumption. The cheese production process requires a lot of blue water in different stages: Raw milk needs to be pre-treated through pasteurization, separation, and standardization, before beginning the actual cheese production. Afterwards come the phases of coagulation, breaking of the curd, cooking, extraction of the curd, shaping, salting, and maturation. Much of the water used during cheese production is dispersed in the form of wastewater and only a part is incorporated into the finished product, from 40–70% depending on the type of cheese [68].

Cheese contributed greatly even to the grey VW of the Trentino Alto Adige dairy system, followed by milk. The grey component provides an indication of the level of pollution generated during the production process. In the case of the dairy supply chain, the increase in the grey water component is mainly due to animal waste, use of fertilizers and pesticides for forage crops, and sediments from eroded pastures [27,69].

All products contribute greatly to the green VW of the Trentino Alto Adige dairy industry (Figure 3). Until it becomes blue water, green water does not contribute to environmental flows, which are needed for the freshwater ecosystem protection, and it is not accessible for other human uses [70]. Although the green VW value is particularly high, it does not contribute *per se* to water scarcity and has a less invasive impact on water resource balances than the blue water consumption [53].

The VW related to consumptive water use includes green and blue VWs [59]. The consumptive VW represents about 87% of the total VW of dairy production in Trentino Alto Adige, with a value of about 200 million m^3^.

In order to assess the water sustainability of dairy production, the consumptive VW was compared with the water availability (WA) of the study area (Figure 1). In 2018, the effective infiltration in Trentino Alto Adige amounted approximately to 16 billion m^3^, allowing for a consistent recharge.

The consumptive VW related to dairy production, represents about 1% of the WA measured by the effective infiltration [61], taking into account that much of the runoff water, given the high steepness of the study area and its distance from the sea, ends up in the catchments, feeding them. Considering the effective infiltration as a water availability measure allows for the obtaining of an overestimated precautionary result. Since effective infiltration represents a flow measure, its comparison with the consumptive VW of dairy products results in a measure of the impact of dairy activities exclusively on the regenerative capacity of water, and not on the actual stock. Therefore, we can conclude that the dairy production of the study area, consuming only 1% of the regenerated water, does not cause water stress in the region.

Although the dairy sector involves generally water-intensive activities along the whole supply chain, Trentino Alto Adige region has hydrogeological and meteorological characteristics suitable for developing such productions without impacting significantly on local water resources, thus ensuring a strong competitive advantage over the other Italian regions in terms of water sustainability.

## 4. Conclusions

As the first application of virtual water assessment to the dairy industry in Trentino Alto Adige, this study demonstrated that dairy products can be produced without great potential to contribute to freshwater scarcity, respecting the hydrological cycle (Figure 2). Thus, the generalization that the growing demand for dairy products is one of the major driving factors for water scarcity is not supported in this case. Since Italy is greatly heterogeneous, the opportunities for water footprint reduction are multiple, and examining the regional differences in water footprints of all major dairy commodities is strongly needed.

In order to reduce the Italian internal and external virtual water, respectively associated with dairy domestic production and imported products from other countries, environmental policies should focus on the development of the Protected Designations of Origin and typical dairy productions, particularly in suitable mountain areas where small-scale farms represent a lively entrepreneurial substrate.

Until today, water has mostly been considered a resource to be managed preferably locally or regionally. This approach does not consider that many water issues are related to remote consumption elsewhere [71]. For dairy products, it is difficult to establish whether they relate to remote water depletion or pollution, because animals are often fed a variety of feed ingredients, whose supply chains are difficult to trace.

In Trentino Alto Adige, this problem is rather irrelevant because most of the traditional dairy products are recognized as Protected Designation of Origin (PDO) and produced by mountain traditional small-scale animal farms. Milk, cheese, and butter, in fact, must originate mandatorily from animals raised locally and that grazed locally or were otherwise fed with locally grown feedstuffs. Notwithstanding, from an economic point of view, the small-scale mountain dairy farms are at a greater disadvantage than intensive farms because of their limited productivity [72]. In this regard, during the last five decades, agriculture and livestock systems in the Alps experienced an important structural transformation: The number of traditional and small farms has been decreasing, and they are being replaced by larger, more modern and specialized farms [73], characterized by intensive livestock and crop productions. This transition, the intensity of land use, and the decline of traditional grazing in the highlands could have negative effects on landscape quality and biodiversity, as well as on water resources. In particular, according to Mekonnen and Hoekstra [5], livestock products from industrial farming systems consume and pollute about 97% more surface and groundwater (blue and grey) than livestock products from pastures or mixed systems.

Moreover, the increasing complexity of the animal product system hides the existing links between the food we eat, the resources used to produce it, and the associated supply chain impacts [74]. Interventions to reduce water footprints, although desirable, should not be taken without considering the potential consequences for other resource impact categories, as well as social and economic factors.

This study can be considered as a basis for further scientific efforts addressed at the evaluation of the dairy production sustainability within particularly suitable areas and regions.

## Figures and Tables

**Table 1:** Data source, classifications, and acronyms.

Macro-Category	HS Code	Data Acquired	Acronym	Data Source
Water Footprint	/	Green Water Footprint (m^3^/ton)	*WF_Green_*	[22]
	/	Blue Water Footprint (m^3^/ton)	*WF_Blue_*	
	/	Grey Water Footprint (m^3^/ton)	*WF_Grey_*	
Dairy Production	040110	Skimmed milk (ton)	*DP*	[58]
	040120	Low-fat milk (ton)		
		Whole milk (ton)		
	040510	Butter (ton)		
	040610	Fresh and soft cheese (ton)		
	040606	Semi-hard and hard cheese (ton)		
Water Availability	/	Precipitation (mm)	*P*	[42]
	/	Real evapotranspiration (mm)	*ET_r_*	
